# Association between serum 25-hydroxyvitamin D and diabetic kidney disease in Chinese patients with type 2 diabetes

**DOI:** 10.1371/journal.pone.0214728

**Published:** 2019-04-24

**Authors:** Shaofeng Xie, Liji Huang, Wen Cao, Yongxin Hu, Hongping Sun, Lin Cao, Kemian Liu, Chao Liu

**Affiliations:** 1 Department of Endocrinology, Jiangsu Province Hospital on Integration of Chinese and Western Medicine, Affiliated to Nanjing University of Chinese Medicine, Nanjing, People’s Republic of China; 2 Department of Endocrinology, Affiliated Hospital of Nanjing University of Chinese Medicine, Nanjing, People’s Republic of China; University of Mississippi Medical Center, UNITED STATES

## Abstract

**Objective:**

The objective was to assess the association between 25-hydroxyvitamin D (25OHD) level and diabetic kidney disease (DKD) in Chinese patients with type 2 diabetes.

**Methods:**

Data pertaining to 351 in-patients with type 2 diabetes were collected. Subjects were classified into three groups based on the level of urinary albumin-to-creatinine ratio (UACR). UACR < 30 mg/g was defined as normoalbuminuria, while UACR levels of 30–300 mg/g and ≥ 300 mg/g were defined as microalbuminuria and macroalbuminuria, respectively. Serum 25OHD and other clinical characteristics among various UACR groups were compared. The relationship between albuminuiria and 25OHD was analyzed.

**Results:**

The prevalence of 25OHD insufficiency in the microalbuminuria group was significantly higher than that in the normoalbuminuria group (25.1% *vs*. 19.6%; *P* < 0.05); patients with macroalbuminuria had the highest prevalence of 25OHD deficiency (37.8%; *P* < 0.01 versus normoalbuminuria). Logistic regression analyses demonstrated that low 25OHD levels were associated with DKD [odds ratio (*OR) =* 1.51, 95% confidence interval (*CI*) 1.16–1.97). The association was more robust after adjusting for sex, hypertension, increased systolic blood pressure, glycemic status, and hyperuricemia (*OR* = 1.62, 95% *CI* 1.19–2.20).

**Conclusions:**

The prevalence of vitamin D insufficiency/deficiency in patients with albuminuria was overtly higher than that in patients without albuminuria among Chinese adults with type 2 diabetes. Vitamin D insufficiency/deficiency was independently associated with DKD in type 2 diabetes.

## Introduction

Diabetes mellitus is a growing worldwide epidemic. In China, an estimated 118.5 million people (10.4% of the adult population) were affected by diabetes in 2013 [1]. Diabetic kidney disease (DKD) is the most common long-term microvascular complication and the leading cause of chronic kidney disease (CKD) and end-stage renal disease (ESRD); globally, DKD accounts for an estimated 20–40% of cases of ESRD [2–3].

DKD is characterized initially by glomerular and tubuloepithelial hypertrophy, thickening of basement membranes, and excess extracellular matrix deposition, which eventually lead to glomerulosclerosis and tubulointerstitial fibrosis. The main clinical manifestations of DKD are progressive albuminuria and decline in renal function [4]. Risk factors for DKD include genetic polymorphisms, long-term hyperglycemia, obesity, hypertension, and dyslipidemia [5–6]. The pathophysiological changes of DKD are likely attributable to the metabolic and hemodynamic abnormalities; however, the exact underlying mechanisms are complex and may involve multiple pathways. Studies have demonstrated that the activation of the intrarenal renin-angiotensin system (RAS) plays a critical role in the causation of progressive renal injury in DKD [7].

Vitamin D and its active metabolite 1,25-dihydroxycholecalciferol (also known as calcitriol) are steroid hormones that help regulate the metabolism of calcium and phosphate, and play an important role in maintaining bone health [8]. The activity of vitamin D is mediated by vitamin D receptors, which are also expressed in almost all cell types in the kidney, including tubular cells, glomerular mesangial cells, and the podocytes [9–10]. In recent years, emerging evidence from clinical and animal studies suggests a prominent protective role of vitamin D in chronic kidney disease [11]. Vitamin D inhibits the activation of RAS by down-regulating renin expression and thus plays a protective role in DKD. Diabetic vitamin D receptor-null mutant mice were shown to develop more severe renal damage due to enhanced RAS activation in the kidney [10–11]. Treatment with Vitamin D and its analogues was shown to improve renal function and confer a significant survival advantage in patients with CKD [12].

However, some studies have yielded inconsistent results pertaining to the relationship between vitamin D status and DKD [13–16]. Therefore, in this study we determined the association between serum 25OHD and albuminuria in Chinese patients with type 2 diabetes. Our finding may provide evidence for the prevention and management of DKD in this population.

## Methods

### 1 Subjects

This cross-sectional study was conducted at the department of endocrinology, Jiangsu Province Hospital on Integration of Chinese and Western Medicine from January to December, 2017. The selection criteria were as follows: (1) hospitalized patients with type 2 diabetes mellitus; (2) age range, 25–75 years.

The diagnostic criteria for diabetes mellitus conformed to the World Health Organization standards (1999) [17]. Type 2 diabetes mellitus was diagnosed by an endocrinologist based on history and elevated blood glucose levels. The exclusion criteria were: (1) fasting blood glucose > 13.9 mmol/L, or glycated hemoglobin A_1c_ (HbA_1c_) > 11.0%, or history of diabetic ketoacidosis or hyperosmotic coma in the preceding 4 weeks; (2) uncontrolled blood pressure (> 180/120 mmHg), evidence of cardiac insufficiency or acute cardiovascular and cerebrovascular diseases; (3) urinary infection, cystic kidney diseases, nephrolithiasis, glomerular or lupus nephritis, or other identified kidney diseases; (4) acute infectious disease or abnormal leukocyte count and neutrophil count in the immediately preceding 2 weeks; (5) serum parathormone (PTH) > 90 ng/L or thyroid stimulating hormone (TSH) > 10.0 μIU/L or TSH < 0.1 μIU/L; (6) patients with osteoporosis or those receiving vitamin D, calcium, or bisphosphonate therapy; (7) severe liver disease, cancer, or blood disorder; (8) body mass index (BMI) < 18.5 kg/m^2^; (9) estimated GFR (e-GFR) < 60 mL/min.

A total of 862 patients type 2 diabetes were eligible for inclusion. Of these, 511 patients were excluded because of following reasons: patients with acute diabetic complications (n = 105); patients with urinary infection, nephrolithiasis, or other known kidney diseases (n = 72); patients with acute cardiovascular or cerebral vascular diseases (n = 45), acute infection or neutrophil abnormalities (n = 64); patients with serum PTH > 90 ng/L or TSH > 10.0 or < 0.1 mU/L (n = 19); patients with osteoporosis or those using vitamin D (n = 36); patients with e-GFR < 60 mL/min (n = 56), and patients for whom complete data was not available (n = 36). Finally, data pertaining to 351 patients were included in the analysis. Our study protocol was approved by the ethics committee of Jiangsu Province Hospital on Integration of Chinese and Western Medicine. Written informed consent was obtained from each participant before data collection.

### 2 Collection of general information

Data pertaining to demographic characteristics, duration of diabetes, personal medical history (including treatment history), history of smoking and consumption of alcohol was collected by trained physicians at admission. Body weight and height were measured without shoes and in light clothing during physical examination. Body mass index (BMI) was calculated as weight (kg) divided by height squared (m^2^). Blood pressure was measured twice at the right brachial artery in sitting poison using electronic pressure monitor (Omron BP-742N; Omron Healthcare Co. Ltd., China) after resting for 5 minutes. The mean of two readings was used for analyses.

### 3 Laboratory measurements

Venous blood samples were obtained between 6:00 Hrs and 8:00 Hrs after overnight fasting for 8–10 hours, and then followed by a random spot urine specimen collection. Lab indices included fasting blood glucose (FBG), lipid profile, blood urea nitrogen (BUN), serum creatinine (Scr), serum uric acid (SUA), calcium, phosphate, HbA_1c_, TSH, and PTH. Serum lipid profile included triglycerides (TG), total cholesterol (TC), low-density lipoprotein-cholesterol (LDL-C) and high-density lipoprotein-cholesterol (HDL-C). Blood biochemical indices were examined using Roche cobas 8000 automatic analyzer (Roche Diagnostics, Shanghai, Ltd.). FBG was detected by the glucose oxidase method. HbA_1c_ level was determined by high-performance liquid chromatography. Urinary albumin-to-creatinine ratio (UACR) in the random spot urine specimen was measured by immune turbidimetry [18]. We determined serum 25OHD concentrations using electrochemiluminescence immunoassay (ECLIA) on an Advia-Centaur-XP immunoassay system (Siemens healthineers, Ltd. China). The intra- and inter-assay coefficients of variation were below 2.1–3.4% and 2.3–3.9%, respectively.

All biochemical investigations were conducted at our hospital laboratory. The chronic kidney disease epidemiology collaboration (CKD-EPI) equation was used to calculate the e-GFR (expressed in mL/min/1.73 m^2^) [19].

### 4 Diagnostic categories

According to the diagnostic criteria in the Application Guidelines for Vitamin D and Bone Health in Adult Chinese [20], the vitamin D status were classified into the following 3 categories based on serum 25OHD levels: vitamin D sufficiency (≥ 50 nmol/L); vitamin D insufficiency (30–50 nmol/L); vitamin D deficiency (< 30 nmol/L). DKD was defined according the level of UACR [21]: values < 30 mg/g were defined as normoalbuminuria, whereas values of 30–300 mg/g and ≥ 300 mg/g were defined as microalbuminuria and macroalbuminuria, respectively. Hypertension was defined as systolic blood pressure ≥ 140 mmHg and /or diastolic blood pressure ≥ 90 mmHg or previous diagnosis of hypertension, or treatment with anti-hypertensive drugs [22]. Dyslipidemia was defined as follows: TC ≥ 5.17 mmol/L, TG ≥ 1.69 mmol/L, HDL-C ≤ 1.30 mmol/L or < 1.03 mmol/L (if female), or LDL-C ≥ 2.6 mmol/L [23]. Three categories for diabetes duration were used: <5 years, 5–10 years, and ≥ 10 years. Based on the age, the subjects were classified as young (<40 years), middle-aged (40–60 years) and elderly (≥ 60 years). Based on the level of HbA_1c_, glycemic status was categorized as good (< 7.0%), fair (7.0%–8.0%), and poor (≥ 8.0%). Based on the levels of symbolic and diabolic blood pressure, blood pressure control was classified as good (< 130/80 mmHg), fair (140–130/80–90 mmHg) and poor (≥ 140/90 mmHg). Estimated GFR was classified as low (≤ 90 mL/min) and normal (≥ 90 mL/min). SUA level was categorized as normal (< 420 μmol/L) and high (≥ 420 μmol/L). Other clinical parameters were categorized based on the upper limit of the normal reference range: TSH ≥ 4.5 μIU/L; PTH ≥ 65 mmol/L; serum calcium ≤ 2.2 mmol/L. Based on the BMI, patients were classified as normal weight (18.5–23.9 kg/m^2^), overweight (24.0–27.9 kg/m^2^) and obese (≥ 28.0 kg/m^2^) according to the Chinese criteria [24].

### 5 Statistical analysis

The EpiData 3.0 software (EpiData Association, Odense Denmak) was used for data entry and processing. All data were obtained independently by two investigators and both databases were rechecked. The SPSS version 18.0 software (SPSS inc., Chicago, IL, USA) was used to perform all statistical analyses. Normally distributed continuous variables are presented as mean ± standard deviation (SD), while non-normally distributed continuous variable are presented as median (interquartile range, IQR). Categorical variables are presented as frequency (percentage). Between-group differences with respect to continuous variables were assessed using the Newman-Keuls method or the Kruskall-Wallis test; those with respect to categorical variables were assessed using the Chi-squared.

The odds ratios (*OR*) and the corresponding 95% confidence intervals (*CI*) for microalbuminuria and macroalbuminuria in relation to vitamin D status were obtained by multiple logistic regression analysis after adjusting for potential confounding variables; normal normoalbuminuria was used as the reference group. An *OR* < 1.0 was regarded as a protective factor. In all analyses, two-sided *P* values < 0.05 were considered indicative of statistical significance.

## Results

### 1 General characteristics

Out of the 351 subjects, 242 (68.9%) were male and 109 (31.1%) were female. The percentage of female was lower because more women with diabetes were excluded due to their susceptibility to urinary tract infection, which influences urinary protein excretion. The mean age of patients was 55.2±10.3 years and the average duration of diabetes was 7.7±6.0 years; 176 (50.1%) subjects were affected by hypertension. In addition, there were 305 patients (86.9%) in G1 stage and 46 patients (13.1%) in G2 stage according to the e-GFR [20].

Out of the 351 subjects, 135 (38.5%) were classified as vitamin D insufficiency (including 52 women), while 85 (24.2%) were classified as vitamin D deficiency (including 31 women). The ratio of vitamin D insufficiency and deficiency in female patients was significantly higher than that in male patients (76.1% *vs*. 56.6%, *P* < 0.01).

### 2 Comparison of clinical characteristics among various UACR groups

The clinical characteristics of the study population disaggregated by various albuminuria groups are described in Table 1; no significant between-group differences were observed with respect to HbA_1C,_ e-GFR, SUA, calcium, TC, HDL-C, LDL-C, PTH, or TSH (*P* > 0.05 for all). Significant differences were observed between the normoalbuminuria and microalbuminuria groups with respect to age, proportion of female patients, patients with hypertension, use of angiotensin converting enzyme inhibitors (ACEi) or angiotensin-receptor blockers (ARBs), SBP, HbA_1C,_ and TG (*P* < 0.05). Patients with macroalbuminuria were significantly older, had increased BMI, longer diabetes duration, more ACEi or ARBs use, elevated SBP and DBP, increased HbA_1C,_ TG and SUA than those with normoalbuminuria (*P* < 0.05).

**Table 1:** Baseline characteristics grouped according to UACR.

Variables	UACR (mg/g)			P-value
	Normo (< 30)	Micro (30–299)	Macro (≥ 300)	
Male/Female	110/33	109/62	23/14	0.014
Age (yr)	53.4±10.6	56.0±9.9	57.6±10.7^a^	0.027
Diabetes Duration				
<5yr (N,%)	56(39.1%)	68(39.8%)	2(5.4%)	
5-10yr (N,%)	35(24.5%)	36(21.1%)	11(29.7%)	0.004
>10yr (N,%)	52(36.4%)	67(39.2%)	24(64.9%)	
Hypertension (N,%)	58(40.6%)	90(52.6%)	28(75.7%)	<0.001
ACEi/ARBs Use (N,%)	35(24.5%)	66(38.6%)	22(59.5%)	<0.001
BMI (kg/m^2^)	25.5±3.7	26.1±3.6	27.6±4.5^a^	0.009
Systolic BP (mmHg)	130.9±14.4	136.8±16.6^a^	141.7±14.7^a^	<0.001
Diastolic BP (mmHg)	79.2±8.9	81.2±10.6	83.8±9.3^a^	0.024
HbA_1c_(%)	8.03±1.46	8.39±1.47	8.50±1.37	0.051
e-GFR (ml/min/1.73m^2^)	101.2±12.3	100.6±14.1	94.5±15.7	0.025
SUA (μmol/l)	320.9±73.0	344.8±89.9	355.4±86.3^a^	0.076
Total Cholesterol (mmol/l)	4.56±1.04	4.64±1.05	4.59±1.19	0.799
Triglyceride (mmol/l)	1.36(0.93–1.85)	1.63(1.08–2.49)^a^	1.62(1.32–2.48)^a^	0.070
LDL-C (mmol/l)	3.0±0.87	3.01±1.03	2.88±1.13	0.856
HDL-C (mmol/l)	1.13±0.34	1.11±0.30	1.12±0.43	0.805
Serum Calcium (mmol/l)	2.29±0.09	2.29±0.10	2.33±0.12	0.109
PTH (ng/l)	39.9±12.5	42.5±15.2	39.2±19.3	0.200
TSH (μIU/l)	2.24±1.23	2.23±1.43	2.25±1.41	0.994
UACR (mg/g)	19.9(15.7–25.3)	55.6(40.6–101.2)^a^	620.3(396.7–1108.8)^a^^b^	<0.001
25OHD	18.85±7.01	17.30±6.30	16.33±6.98	
>50 nmol/L	67(46.8)	53(31.0)	11(29.7)	
30~50 nmol/L	48(33.6)	75(43.9)^a^	12(32.4)	0.003
<30 nmol/L	28(19.6)	43(25.1)^a^	14(37.8)^a^	

^a^*P* < 0.05 compared with UACR of < 30 mg/g

^b^*P*<0.05 compared with UACR of 30–300 mg/g

Abbreviations: ACEi, angiotensin converting enzyme inhibitor; ARBs, angiotensin II receptor blockers; BMI, body mass index; BP, blood pressure; HbA_1c_, glycated hemoglobin A_1C_; e-GFR, estimated glomerular filtration rate; SUA, serum uric acid; LDL-C, low density lipoprotein cholesterol; PTH, parathyroid hormone; TSH, thyroid-stimulating hormone

Notably, the percentage of patients with vitamin D deficiency in the microalbuminuria group was significantly greater than that in the normoalbuminuria group (25.1% *vs*. 19.6%, *P* < 0.05). The macroalbuminuria group showed the highest prevalence of vitamin D deficiency (37.8%, *P* < 0.01 versus normoalbuminuria group).

### 3 Logistic regression analysis: risk factors for albuminuria in type 2 diabetes

In the univariate logistic regression analysis, albuminuria was used as the dependent variable and the other clinical parameters were used as independent variables. Female sex, age, BMI, hypertension, raised systolic BP, diastolic BP, glycemic status, hypertriglyceridemia, HDL-C level, and hyperuricemia were risk factors for albuminuria (*P* < 0.05 for all). Low 25OHD levels were risk factors for albuminuria (*P* = 0.002). The association between albuminuria and vitamin D status was analyzed by multivariate logistic analysis. The results showed a significant association between low 25OHD levels and DKD in patients with type 2 diabetes (OR: 1.51, 95% *CI* 1.16–1.97); the association was more robust after adjusting for sex, hypertension, raised systolic blood pressure, glycemic status, and hyperuricemia (OR: 1.62, 95% *CI* 1.19–2.20) (Table 2).

**Table 2:** Logistic regression analysis of albuminuria risk factors in T2DM.

Parameter	Univariate analysis			Multivariate analysis		
	*OR*	*95%CI*	*P*-value	*OR*	*95%CI*	*P*-value
Gender	1.77	1.14–2.75	0.011	1.71	1.03–2.86	0.040
Age	1.03	1.01–1.05	0.007	-		
BMI	1.49	1.12–1.98	0.006	-		
Hypertension	2.14	1.42–3.23	<0.001			
Systolic BP status	1.59	1.26–2.01	<0.001	1.70	1.29–2.23	<0.001
Diastolic BP status	1.42	1.09–1.86	0.009	-		
Glycemic status	1.39	1.09–1.79	0.009	1.52	1.12–2.07	0.007
Hypertriglyceridemia	1.67	1.11–2.52	0.015	-		
HDL-C status	0.63	0.42–0.95	0.027	-		
Hyperuricemia	1.75	1.04–2.96	0.037	1.92	1.36–2.73	<0.001
e-GFR < 90 ml/min/1.72m^2^	2.18	1.28–3.72	0.004	2.20	1.19–4.06	0.012
Low 25OHD levels	1.51	1.16–1.97	0.002	1.62	1.19–2.20	0.002

Abbreviations: BMI, body mass index; BP, blood pressure; OR, odds ratio; CI, confidence interval

## Discussion

In humans, vitamin D from dietary sources or that synthesized in the skin is first converted in liver to 25OHD, which is the predominant circulating metabolite; 25OHD is commonly used as an indicator of vitamin D status in the body. Subsequently, 25OHD is further hydroxylated by 1α-hydroxylase in the renal proximal tubule to 1,25-dihydroxyvitamin D, which is the biologically active form of vitamin D. Due to aging, dietary changes, reduced outdoor activities, low sun exposure, and other causes, vitamin D deficiency or insufficiency is quite common worldwide; an estimated 50%–80% of the total population is affected by vitamin D deficiency or insufficiency [25]. Surveys conducted in Chinese cities located at different latitudes have shown the prevalence of vitamin D deficiency or insufficiency in the population [26–28]. De Boer et al reported that lower 25OHD levels were associated with an increased prevalence of albuminuria in a large representative sample of US adult population with diverse age, race/ethnicity, and diabetes status [29]. Age-related decline in renal function has been shown to be associated with decline in the synthesis, metabolism, as well as the transport of 1,25-dihydroxyvitamin D; patients with ESRD have a higher prevalence of vitamin D deficiency [30–33].

In our study, 86.9% patients had normal or elevated e-GFR. Out of the total 351 subjects, 62.7% were affected by vitamin D deficiency or insufficiency. In two Chinese cross-sectional surveys among diabetic inpatients, the proportions of subjects with 25OHD levels < 25 mmol/L and < 50 nmol/L were 59.7% and 83.5%, respectively [34–35]. These findings demonstrated that vitamin D deficiency or insufficiency is quite common among Chinese patients with type 2 diabetes. Moreover, greater prevalence of vitamin D deficiency or insufficiency is observed in women, which is consistent with several epidemiologic surveys [36–37]. It is speculated that more indoor work, less outdoor activities, and less sun exposure among women are possible causes. Further, patients with microalbuminuria had a higher prevalence of vitamin D insufficiency or deficiency compared to those with normoalbuminuria; in addition, patients with macroalbuminuria had more severe vitamin D insufficiency or deficiency. Multivariate regression analysis showed that gender, hypertension, increased systolic blood pressure, poor glycemic control, hyperuricemia, and low 25OHD levels were independent risk factors for albuminuria in type 2 diabetic patients. Of note, type 2 diabetes patients with low vitamin D levels were at a 62% higher risk of albuminuria as compared to vitamin D sufficient patients. Adjustment for potential confounders did not attenuate this association, which suggests that low vitamin D level may be a risk factor for albuminuria, an established and modifiable risk factor for kidney disease in the general population. These findings are consistent with those of a previous Chinese cross-sectional survey that showed a close relationship between the serum 25OHD levels and UACR level in patients with type 2 diabetes [35].

Epidemiology of Diabetes Interventions and Complications (EDIC) study [13] is an observational follow-up study of the Diabetes Control and Complications Trail (DCCT), which observed 1193 participants with type 1 diabetes over a period of up to 16 years. The study found that 25OHD deficiency was associated with a 65% higher risk of albuminuria compared with sufficient 25OHD status; however, 25OHD deficiency was not associated with decreased e-GFR. Another Japanese study enrolled 442 patients with type 2 diabetes, of which, 50 patients were at CKD stage 1, 198 at CKD stage 2, and 194 at CKD stages 3–5. They found that female sex, increased age, and proteinuria were the main risk factors for low vitamin D levels [14]. The conclusion was consistent with our study. However, in two longitudinal observational studies, 289 type 2 and 220 type 1 diabetic patients were followed up for a median of 15.0 (0.2–23) years and 26 (1.0–29) years, respectively. Joergensen, et al [15–16] found that severe vitamin D deficiency increased the risk of all-cause mortality, but not the development of DKD. The inconsistent findings may be due to differences of research methods, inclusion criteria of subjects, racial differences, or other confounders.

In this study, the duration of diabetes in patients with macroalbuminuria was significantly longer than that in patients with normoalbuminuria and microalbuminuria. However, on multivariate analysis, duration of diabetes was not an independent risk factor for albuminuria. This is likely attributable to the wider use of ACEi and ARBs for treatment of hypertension in patients with type 2 diabetes. Studies have shown that early use of ACEi and ARBs can effectively relieve proteinuria and attenuate progression of renal injury by lowering glomerular hyperfiltration in diabetic patients; however, the effect of these drugs is quite weak once the patients develop macroalbuminuria [38–39].

A number of epidemiologic studies have reported SUA as an independent risk factor for renal disease in diabetic patients [40–42]. Our results are in line with these previous studies. We found a positive association between SUA level and albuminuria in patients with type 2 diabetes. However, the effects of uric acid on the progression of renal disease are still not fully understood.

DKD is characterized by the appearance of persistent clinical albuminuria and reduction in the GFR. Epidemiological studies have shown that about 25–40% of patients with type 1 and type 2 diabetes develop clinical proteinuria, which eventually progresses to ESRD [1]. Therefore, besides long-term hyperglycemia, there are other risk factors that contribute to the development of DKD. Several possible mechanisms may explain the link between vitamin D and nephropathy in diabetes. Diabetic vitamin D receptor knockout mice were shown to develop multiple pathophysiological changes that eventually resulted in proteinuria and renal injury [43]; these changes included thickening of glomerular basement membrane, loss of podocytes, and greater production of renin and angiotensinogen. Secondly, in a study by Thraikaill et al [44], greater renal excretion of vitamin D-binding protein in patients with DKD was shown to result in secondary decrease in vitamin D level. Third, many lines of studies have implicated kidney inflammation as a cardinal pathogenetic mechanism of progression of DKD [45]. Jablonski et al [46] found that lower 25OHD status was associated with vascular endothelial dysfunction among healthy middle-aged and older adults, and that was mediated in part by nuclear factor κB-related inflammation. Finally, oxidative stress caused by hyperglycemia is considered a common and important pathogenetic mechanism for vascular complications in diabetes [47]. In a study by Deng et al. [48], vitamin D (calcitriol) was shown to markedly alleviate kidney injury by attenuating renal oxidative stress in diabetic rats.

Some limitations of our study should be acknowledged. Firstly, this was a cross-sectional study; we did not assess long-term follow-up data pertaining to vitamin D levels and proteinuria. Our study only identified the association between vitamin D and proteinuria in patients with type 2 diabetes. Given the single-center scope of our study, our findings may not be generalizable to the entire Chinese population. Secondly, repeated measurements of UACR, which are recommended to increase specificity were not available. Finally, some potential confounders such as sun exposure, outdoor sports, smoking status, nutritional status, and season of vitamin D detection, were not included in the analysis.

In conclusion, the results of our study show that low vitamin D level is a major predictor of incident nephropathy in type 2 diabetic patients. Further, large-scale surveys and prospective cohort studies are required to clarify the causal relationship between vitamin D and protainuria and to elucidate the scientific basis for the prevention and treatment of DKD.

## Supporting information

S1 File Manuscript 351diabetes.XLS file including demographic and experimental data of 351 patients with type 2 diabetes for this study. (XLS)
